# Hierarchical organization of human physical activity

**DOI:** 10.1038/s41598-024-56185-0

**Published:** 2024-03-12

**Authors:** András Búzás, András Makai, Géza I. Groma, Zsolt Dancsházy, István Szendi, Laszlo B. Kish, Ana Raquel Santa-Maria, András Dér

**Affiliations:** 1 Institute of Biophysics, HUN-REN Biological Research Centre, Temesvári Krt. 62, P.O.B. 521, Szeged, 6701 Hungary; 2 Department of Psychiatry, Kiskunhalas Semmelweis Hospital, 1 Dr. Monszpart László Street, Kiskunhalas, 6400 Hungary; 3 Department of Electrical and Computer Engineering, Texas A&M University, TAMUS 3128, College Station, TX 77843-3128 USA; 4 Wyss Institute for Biologically Inspired Engineering, Harvard University, Boston, MA USA

**Keywords:** Biophysics, Physiology, Psychology, Health care, Medical research, Mathematics and computing, Physics

## Abstract

Human physical activity (HPA), a fundamental physiological signal characteristic of bodily motion is of rapidly growing interest in multidisciplinary research. Here we report the existence of hitherto unidentified hierarchical levels in the temporal organization of HPA on the ultradian scale: on the minute's scale, passive periods are followed by activity bursts of similar intensity (‘quanta’) that are organized into superstructures on the hours- and on the daily scale. The time course of HPA can be considered a stochastic, quasi-binary process, where quanta, assigned to task-oriented actions are organized into work packages on higher levels of hierarchy. In order to grasp the essence of this complex dynamic behaviour, we established a stochastic mathematical model which could reproduce the main statistical features of real activity time series. The results are expected to provide important data for developing novel behavioural models and advancing the diagnostics of neurological or psychiatric diseases.

## Introduction

Actigraphy, a non-invasive tool based on the detection of forearm acceleration by a wristwatch-size recorder, has recently been introduced for measuring the time course of Human physical activity (HPA)^1–4^. Activity signals carry information about the biological rhythms, daily routine, jet lag, and various psychiatric disorders^5^. While some of their characteristics on the circadian scale are already routinely used in medical therapy, the complex structure of the activity patterns on different time scales remains unexplored.

Rhythmic dynamism in time is a fundamental feature of cell and tissue organization maintained against entropy increase, according to the laws of thermodynamics. Living organisms are thermodynamically open, almost always operating near equilibrium, and naturally subject to disturbance. At the same time, they exhibit complex spatial and temporal regularities, including coordinated automatic behaviour. Chronobiology science also tries to reveal, on the one hand, the molecular processes within the cells that cause rhythmic behaviour in time, and also the mechanisms that ensure the spatial spread of communication between cells. In the background of rhythm generation, the role of opposing activation and inhibition can be significant^6^, moreover, activation can prevail in the short term, while inhibition can prevail in the longer term^7^.

Concerning the role of environmental factors in biological rhythms, there are firm experimental evidences that there is a physicochemical connection between rhythms in the geosphere and the biosphere^8^. It is assumed that geological oscillations synchronize biological ones: low-frequencies impact on population dynamics, while higher frequencies on individuals, as the latter is evidenced, e.g., by the most well-studied circadian rhythms. The ultradian band was the only frequency range where the correlation between the geological and biological frequencies was not obvious^9^.

Rhythms shorter than 24 h are called ultradian^10^, and more often we mean activities lasting from a few minutes to 6 h^11,12^. Oscillations shorter than a second are characteristic of the periodicity of the electrical activity of the brain and heart. Living tissues and cell cultures show ultradian rhythmicity in the size of cells, the activity of protein synthesis and enzymes, the production of ATP and many hormones, and the activity of cellular respiration^13^. At the level of the organism, the temperature of the body and organs, CO_2_ production, O_2_ consumption, blood pressure, hormone secretion, digestion, urine and stool excretion, and sleep phases^13,14^ also follow ultradian rhythms.

**Figure 1:**
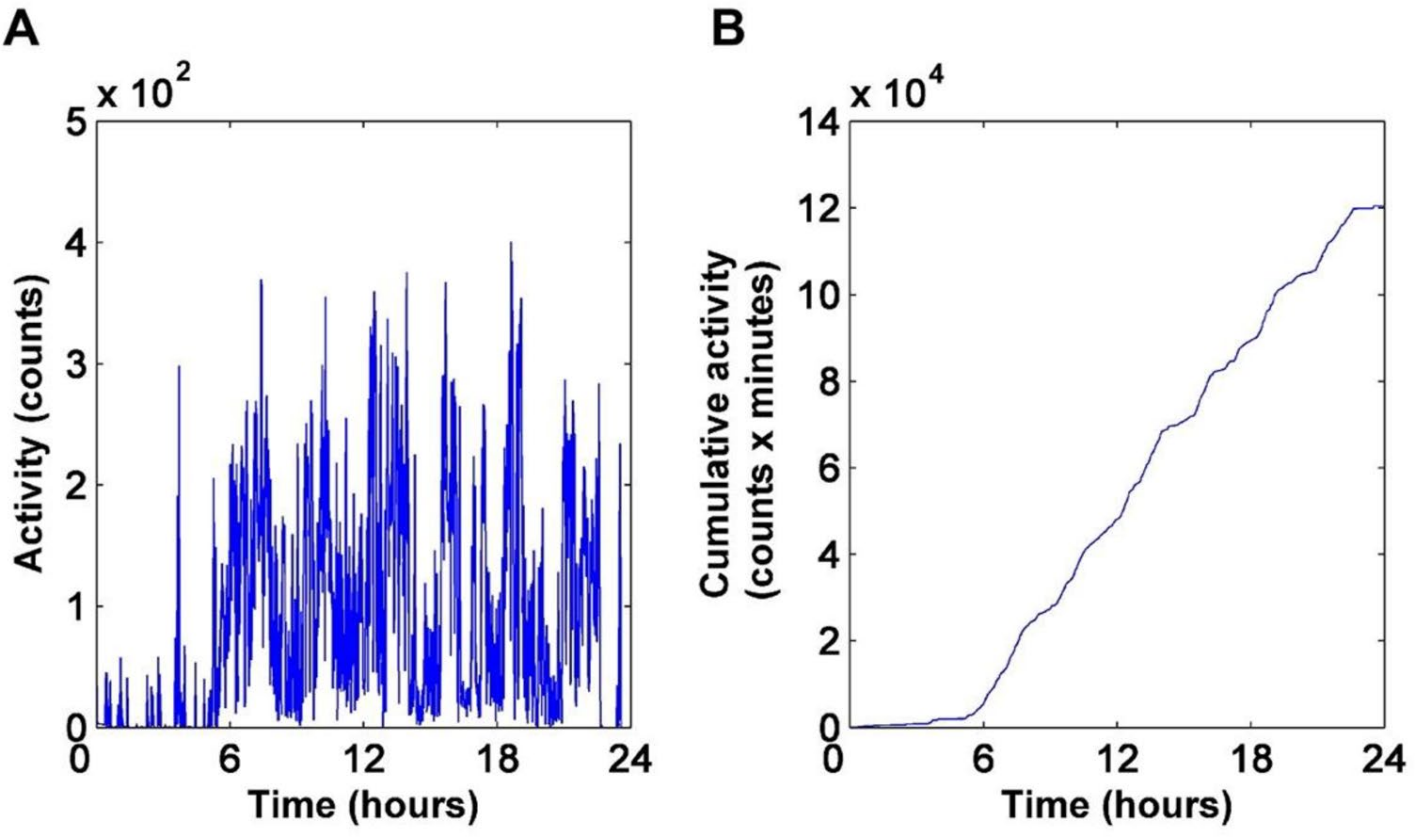
(**A**) A typical daily activity recording taken by an actigraph. Acceleration values were recorded by 40 Hz sampling frequency, and the integration time was 1 min. for each depicted data point. (**B**) The running integral function (cumulative sum) of the time series in (**A**).

Since these phenomena are actually often aperiodic, it is often referred to them with the terms 'episodic ultradian events'^9^. This is also manifested in the fuzzy appearance of human physical activity signals. Diurnal activity recordings appear as a fluctuating time series showing strong stochastic features (see, e.g., Fig. 1A). In principle, fluctuations may arise both from exogenous and endogenous origins: the fluctuating environment on the one hand, and nonlinear, possibly chaotic mechanisms on the other. It is a key open question in life and behavioural sciences, whether the stochastic dynamics of physiological rhythms is an essential feature for their function, or it is merely a consequence of environmental fluctuations^15^. Former reports suggest an intrinsic origin of fluctuations in HPA recordings of healthy subjects and demonstrated fractal characteristics in the short-time behaviour of actograms^16,17^.

Back to the early ‘90 s, Rossi et al., established that an 80–90/110–120 min periodicity of active phases was accompanied by rest periods of ca. 20 min, either in the sleep phases, or in active behaviour (see for instance^18^). Physiological data did accumulate too, pointing to crucial periodicities that evidently influence activity: in addition to a 24-h circadian rhythm in the HPA axis hormones, ultradian rhythms of ACTH and cortisol have been identified, most frequently 90–110 min (for instance^19^).

Similarly, the investigation of human infants, coupled with the recognition of the REM (rapid eye movement) sleep cycle, prompted Kleitman to develop the concept of the basic rest activity cycle (BRAC). This theory suggests that the REM sleep pattern persists throughout the entire 24-h day, and it is reflected in other physiological and mental functions^20^. Blessing and Ootsuka^9^ measured several physiological factors, such as body and brain temperature, actigraphy signals, or the temperature of brown adipose tissue (BAT) at the same time in Sprague–Dawley rats that were awake and moving freely. They proved the non-stationary, stochastic nature of ultradian events, and at the same time established that the different measured parameters were synchronized to each other. The latter phenomenon implied a central-nervous-system control of ultradian events, probably distinct from the suprachiasmatic nucleus responsible for the circadian rhythms^9^. In fact, Gerkema et al.^21^ showed that in the hypothalamus, rhythmic patterns of GnRH (gonadotropin-releasing hormone) can be identified in both the preoptic area and the arcuate nucleus. Additionally, in voles, the ultradian feeding oscillator relies on the retrochiasmatic area^10,22^. At the network level, it was identified that midbrain dopaminergic neurons play a key role in the ultradian oscillatory operation of the locomotor periodontium. However, it is not yet known whether these are global generators, what their relationship is with other parameters of the ultradian rhythm at the organizational level (e.g. body temperature and hormone secretion), and what cellular and network mechanisms play a role in generating fluctuations in dopamine activity^23^.

On the other hand, purely internal—however stochastic—pacemakers cannot explain ultradian events, but obviously, external environmental and social factors should also have prominent effects. Note, e.g., that in animal models a fluctuations of weak magnetic field had a modulatory effect on body temperature^8,24,25^, or, in the absence of relevant geophysical cycles, interaction with conspecifics can lead to synchronization of physical activity^10^.

Concerning the physiological role of ultradian events, one might conclude that, contrary to longer, circadian and infradian, rhythms that serve to adapt to predictable changes in the environment, they are believed to ensure readiness for unpredictable changes in adaptation. On these grounds, we assume that the interplay between internal and external driving forces controlling human daily physical activities is manifested in a non-random, hidden structure of ultradian actigraphy signals. However, the main tool used nowadays for evaluating actograms is still Fourier analysis, which—though effective in revealing periodic components^26^—normally fails to identify stochastic structures that seem to be prevalent on the ultradian scale.

In order to reveal the presumed, but hitherto unexplored, non-periodic structure of the organisation of human daily activities, in our analysis, two more adequate statistical methods were used: the probability density function (PDF) and the continuous wavelet (CW) transformation tools. Our PDF analysis clearly showed that diurnal HPA time series have non-random features, attributed to the appearance of activity bursts of similar average intensity (“quanta”), followed by resting phases on the minutes scale, associated with task-oriented actions of the daily routine. The time distributions of active and passive periods are different, the former follows a lognormal, while the latter a power-law distribution. Our CW-analysis, on the other hand, revealed that quanta are organized into superstructures on the hours’ scale, indicating a hierarchical organization of the diurnal human physical activity. To interpret these findings, we developed an integrate-and-fire type stochastic model that could decently reproduce all the main features of the HPA signals recorded during daily routine activities. The results are discussed in the context of former behavioural studies, and their possible implications to psychiatric diagnostics are envisioned.

## Results and discussion

### Statistical analysis of experimental results

A typical daily activity recording is shown in Fig. 1A, demonstrating the most obvious feature of actograms, namely their „fuzzy” appearance due to strong fluctuations.

**Figure 2:**
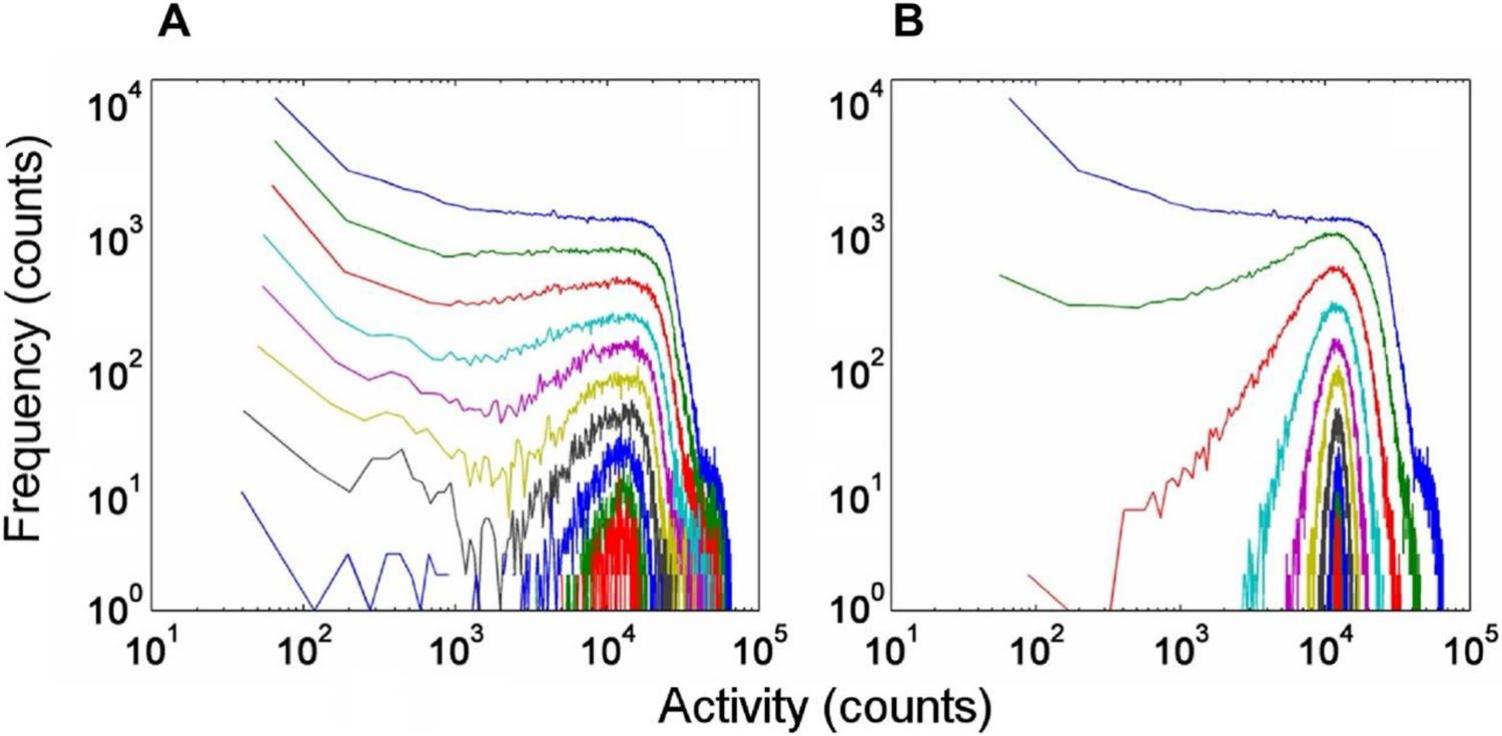
Probability density functions of activity spikes recorded under conditions described in Fig. 1. Curves in (**A**) and (**B**) are obtained from the original daytime activity recordings and the scrambled control, respectively. The time scale of the daily recordings was divided into equal intervals (“boxes”), and activity values were averaged within each box, respectively. PDF functions were calculated from these averaged values. Data are shown for some characteristic, quasi-exponentially distributed box lengths (1, 2, 5, 11, 22, 45, 90, 180, 360, and 720 min) distinguished by different colours (blue, green, red cyan, magenta, yellow, black, blue-green, red), respectively.

In order to elucidate whether the activity signals have a kind of internal stochastic structure, we applied a simple PDF analysis. Data collected from 4 volunteers for an average of 3 weeks all agree in their main features (see also Figs. S1–S3). The basic concepts are presented via a case study, using the analysis of a data set recorded on a single subject for 600 days. Our main focus was on the daytime actograms associated with daily routine activities, so the nights were cut out from the recordings, and analysed later separately. The frequency distribution of the activity values is depicted in Fig. 2A, at different time resolutions. The cut-off in the PDF curves between 10^2^ and 10^3^ counts shows that such high-activity spikes are rare. In Fig. 2B, as a control, we show the same analysis of a virtual time series obtained by randomly mixing the original recording data in time (“scrambling”). The uppermost curves in Fig. 2A,B, corresponding to the shortest (1 min) box length, naturally coincide. However, at longer time intervals the differences become obvious, which indicates the existence of non-random structures in the original HPA signals.

The shapes of the first few traces from the top in Fig. 2A are very similar, in agreement with a previous work^16^ reporting a scale-invariant behaviour of HPA signals for the 30-s to a 6-min time range, as a possible indicator for their chaotic origin. Our results indicate, however, that starting at time windows of a few minutes, the original shape of the PDF is gradually distorted, showing the violation of scale invariance in this time regime. An explicit depression, separating two peaks, is built up in the middle of the curve by increasing the box size, persisting for box lengths of several hours. The meaning of this feature becomes obvious by inspecting the integral function of the daytime activity trace of an average day, where a definite stairway-like structure is observable (Fig. 1B). Apparently, there are two characteristic slopes on this function, which can be associated to the two separate peaks in the PDFs in Fig. 2, located above and below ca. 20 counts/min, respectively. During the higher-activity periods, the average activities are similar, and centred to a higher value (right peak in Fig. 2A, or the higher slope in Fig. 1B). However, it is easy to distinguish resting periods with activities close to zero (left peak in Fig. 2A, or plateaus in Fig. 1B). In other words, on this time scale HPA is quantized: it is distributed such that distinct active periods (“quanta”) are followed by passive ones, and it is quasi-binary, this means that it approximately operates as a “two-gear” machine, either with resting or with a traveling pace.

**Figure 3:**
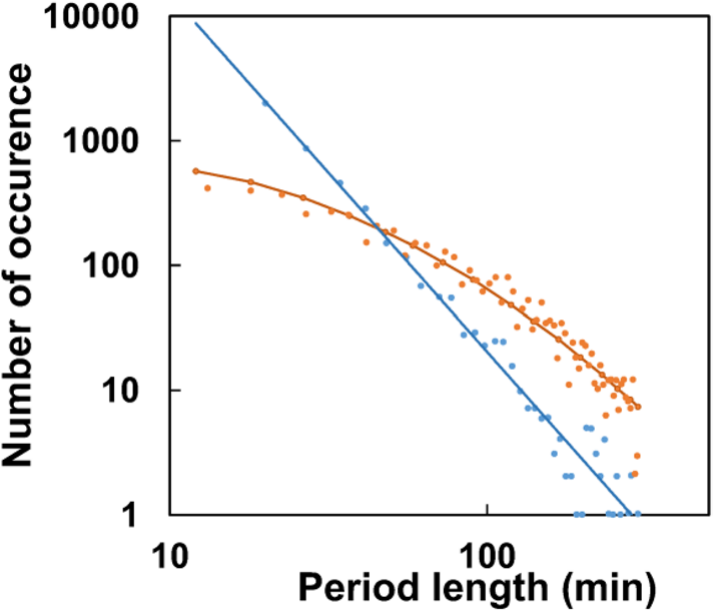
Log–log plots of probability density functions of the lengths of active and passive periods the original daytime recordings were divided into (orange and blue symbols, respectively). A period was considered active if its average activity was higher than 20 counts/min, and passive otherwise. Active PDFs were fitted by lognormal distributions defined by the conventional μ and σ parameters (μ_act_ = 3.5, σ_act_ = 1.3), while the linear decay of the passive PDF values in log–log representation clearly indicates a power-law function (slope ≈ − 2.67).

Figure 3 shows the PDF of the lengths of active and passive periods during the daily routine. The maximum can be found at small values (a few minutes), this means that short intervals are in majority, and the probability of longer ones gradually decreases. The average length of active periods is considerably greater than that of passive ones, however, periods longer than 50 min are rare for both cases. The probability of periods longer than 4 h is less than 10^−3^. Since the few-minutes to approximately 1-h scale is the typical time scale of perceptually salient activities of everyday life (e.g., making a bed, doing the dishes, etc.)^27^, we associate the activity quanta revealed by our analysis with such task-oriented actions.

PDFs of the lengths of the active periods can be asymptotically well-approximated with lognormal distribution, showing that they are determined by a *product* of many small independent factors. The lognormal distribution is commonly used to model, e.g., the lifetime of mechanical units whose failure occurs when the impact of subsequent elementary stress effects reaches a certain threshold (fatigue limit)^28^. Analogously, we infer that complex human activities (related to task-oriented actions) can also be considered because of chains of elementary processes, representing a critical amount of physical and mental effort, required to accomplish a task. This conclusion strongly supports a new, central workspace hypothesis on cognitive actions^29^, implying that for complex tasks, conscious processing consists of multiple serial stages of stochastic accumulation of evidence, until a decision threshold is attained^30^.

On a lower level of the hierarchy of human physiological signals, the lognormal distribution is also observed in the firing rate of a single neuron, which has recently been shown to be the consequence of fluctuations in the charging ion current^31^. On the other hand, the wide occurrence of lognormal distribution of firing rates of neural populations has also been interpreted as a consequence of the highly complex nature of nervous systems^32^ and a Hebbian mechanism of intrinsic learning^33^.

Contrary to the lognormal distribution observed for the lengths of the active periods of human physical activity, the resting times follow a power-law distribution, another typical dynamic feature of complex, hierarchical network structures^34^ (Fig. 3). Note that a similar observation has been made earlier for the statistics using an ad-hoc threshold for raw actigraphy signals, as well^35^.

**Figure 4:**
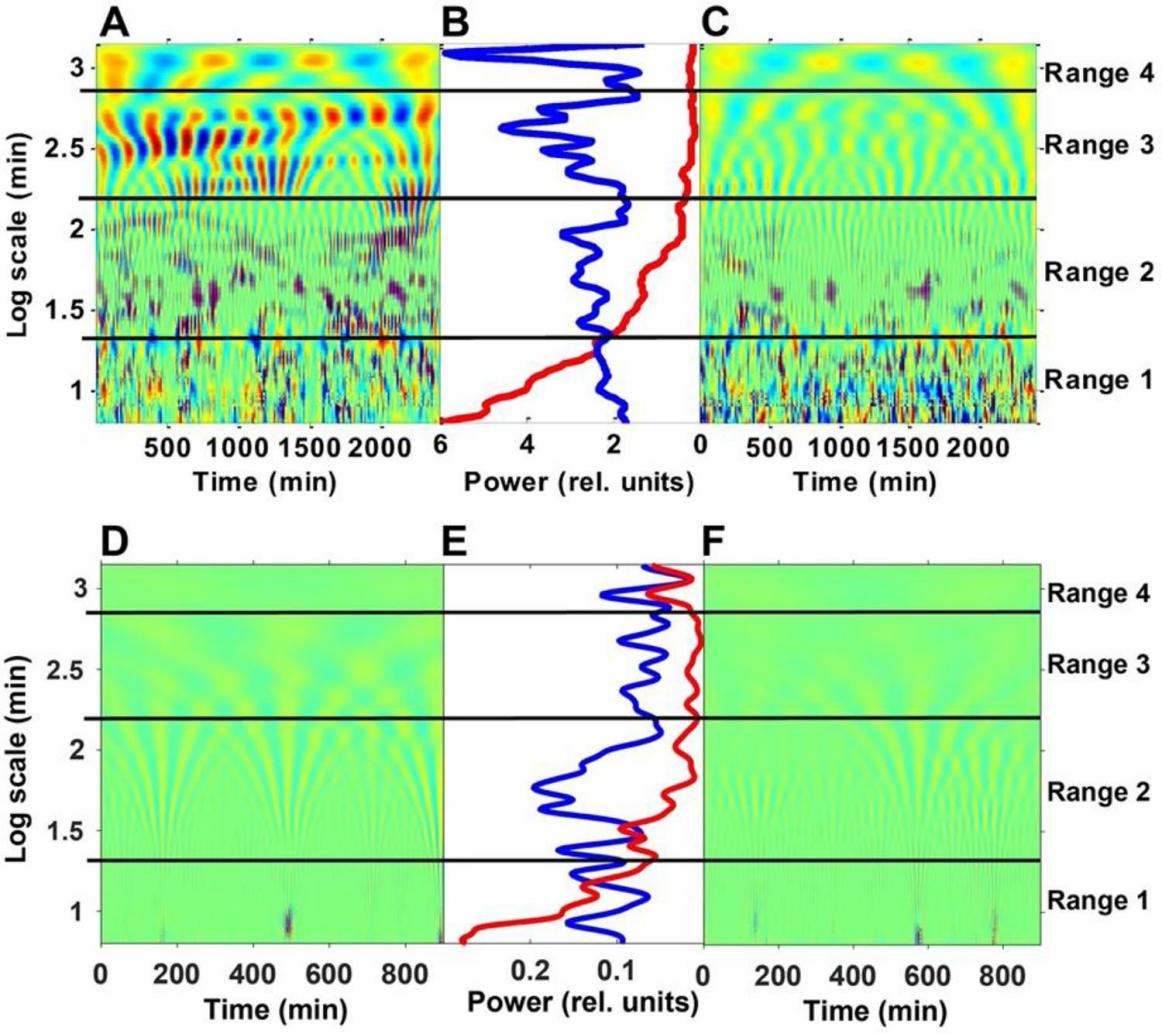
Multiscale representation of the daytime activity recordings. (**A**) Continuous wavelet decomposition of the motion activity signal corresponding to the concatenated wakeful periods of three subsequent days based on the Morlet wavelet. For a better visual demonstration, the wavelet coefficients were divided by $\sqrt s$, where $s$ is the scale parameter to ensure the scale-independent weight of the different patterns^36^. Deep red and deep blue patches correspond to domains of maximal positive and minimal negative coefficients (1 and − 1, respectively), while green colour corresponds to values close to zero. (**C**) The result of the same wavelet decomposition if the individual data points within each period were randomly scrambled to remove temporal correlations. (**B**) Distribution of the square power of the wavelet coefficients over the scale dimension, calculated from the data of 10 subsequent days (blue: original, red: scrambled data). The three horizontal lines separate four well-distinct time-window ranges (Ranges 1–4, corresponding to intervals of ca. 1–20 min, 20 min–3 h, 3–10 h and > 10 h, respectively) of the most typical activity patterns, distinguished via the comparison of the two curves in (**B**). Inserts (**D**), (**E**) and (**F**), respectively, show the results of an analogous evaluation procedure of the corresponding sleep data comprising a concatenated nocturnal time series of 10 successive days. (**D** and **F** are the CW-maps of the original and scrambled data, while E shows the distribution of the square power of the wavelet-coefficients: blue: original, red: scrambled control).

In order to get a deeper insight into this behaviour and reveal any possible hierarchical structure of the actigraphy signals, we applied a continuous-wavelet analysis to the activity recordings. Wavelets are useful tools for analysing periodic or stochastic time series, carrying information both about their time and frequency characteristics^36^. Figure 4A shows the 2D colour plot of a continuous-wavelet (CW) decomposition of daytime activity recordings of 10 successive days (for details, see “Methods”). The x and y axes measure the running time and the scale parameter of the wavelet, respectively, while colours represent correlation coefficients between the wavelet and activity records. In this representation, zones of alternating red and blue colours are related to high structuredness of the activity signal. In accordance with the result of our PDF analysis, one can easily see the main differences between the characteristic features of the wavelet analysis of the activity recording (Fig. 3A) and its scrambled control (Fig. 3C).

Based on the comparison of the power plots of the recording and the control (Fig. 4B), one can distinguish four-time windows (Range 1–4). At time windows of ca. 10 min, the power plots of the original recordings and the control cross each other, while at time windows between ca. 50 min and 4 h, there is an explicit depression in the curve representing the original recording (Fig. 4B, blue line). The corresponding three bifurcations are apparent from the colour pattern of the CW activity graphs (Fig. 4A,C), as well. For scales of less than 10 min (Range 1) we can find a well-distinguished fine structure in the CW graphs of both the original recording and the control. While, in the control these high-frequency patterns are predominant, in the CW plot of the recording structural features are shifted to lower frequencies (higher scales, Fig. 4A,C). In the scale window between^14^ 10 min and 50 min (Range 2), a structure of vertical stripes appears, correlated to the emergence of active and passive periods found in the PDF analysis in the corresponding time regime. Since we know from our PDF-analysis, that active and passive periods longer than 50 min are very rare (Fig. 3), the different structural patterns appearing between 50 min and 4 h in the CW-analysis (Range 3, Fig. 4) must represent another level of hierarchical organization. This should correspond to superstructures into which the activity bursts of Range 2 are organized. Similarly, Range 4 represents the natural, circadian superstructures wherein the bunches of bursts of Range 3 are organized.

In conclusion of the PDF and CW analyses, activity bursts in the daytime recordings seem to be organized into several hierarchical levels. The characteristic activity patterns in the above ranges indicate different underlying physiological processes. In Range 1 (less than 10 min), elementary wrist motions take place. Considering that the time resolution of our measurements (1 min) is considerably longer than the estimated reaction times for preconscious or conscious actions (several 100 ms)^37^ we infer that already these elementary spikes are under, at least partial, voluntary control, nevertheless, their temporal distribution still shows chaotic features^16^. The activity quanta appearing in Range 2 (between ca. 10 min and 50 min) shows that the human actions on this time scale are organized into well-defined work packages that we assigned to task-oriented actions. Because of their stochastic distribution, up to now, this quantum-like behaviour has remained hidden for the conventional evaluation tools used in actigraphy, seeking periodic (e.g. circadian or ultradian) patterns synchronized to the calendar time. At the same time, it indicates that characterization of HPA simply with the familiar total daily count of activity^38^ could easily lead to misinterpretation of the data. Unlike what is generally expected, a higher total daily count does not mainly correspond to an increased amplitude of activity, but rather to a higher duty cycle of bursts of essentially equal amplitudes. The appearance of superstructures in Range 3 (the hours’ scale) indicates an even higher hierarchical level of organization in the daily HPA, since uninterrupted active or passive periods—making up patterns in Range 2—on this time scale are rare (see Fig. 3). As it is expected, on the long end of the investigated time window regime (Range 4), periodic structures occur when approaching the circadian scale, as a limiting frame for the intrinsic and extrinsic rhythms of strong stochastic nature appearing on the shorter time scales.

To reveal whether the sleep data show similar characteristics, as well, we analyzed them separately. The use of actigraphy in sleep medicine is prevalent^5,39^, and it is considered a valuable tool, e.g., in polysomnography^40^. However, unlike most of the related studies, here we were focusing rather on the possible existence of nocturnal rhythms than on other usual measures like the sleep length or fragmentation index^41^. Hence, an evaluation of the nocturnal time series analogous to that of the corresponding daytime data was performed by CW-analysis (for more details, see “Methods”). Although, the data series showed rather sparse and stochastic features, being the average nocturnal activity smaller than the daytime one by more than an order of magnitude, the wavelet-analysis produced decent results: the difference between the maps of the original data and the scrambled control revealed the existence of non-random patterns in the former (Fig. 4D–F).

For better comparability with the daytime results, maps of the sleep data are depicted on the same time-window scale. It is conspicuous that non-random features prevalently appear in Range 2 (between ca. 20 and 100 min), in agreement with our former results established for a different set of subjects^41^. Although the appearance of these events is fuzzy, they might well obey the “20 min—100 min rule”^18^. Contrary to the daytime maps, however, there are no marked structures found in Ranges 3 and 4, implying the lack of higher hierarchical organization. Corresponding CW-maps of the other volunteers show rather similar features (along with that at one of them, the structured part is somewhat upshifted) (Fig. S4).

Altogether, we found distinct non-random patterns in both the daytime and sleep activities, however, their stochastic appearance dominates over their rhythmic features on the ultradian scale.

### Stochastic model

**Figure 5:**
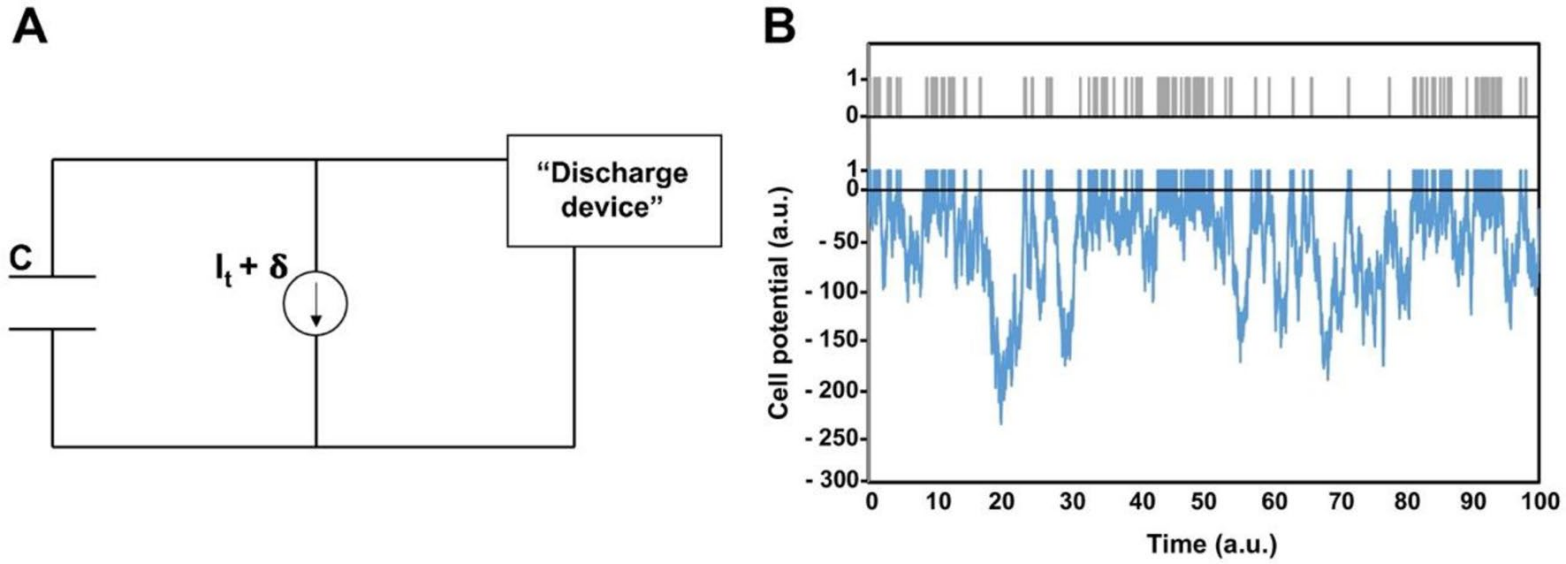
Demonstration of the occurrence of elementary activity bursts by an integrate-and-fire electric-circuit model. (**A**) Capacitor (C) is charged by a constant current (I_t_) superimposed to a Gaussian white noise of half-width δ, and a discharge takes place when the cumulative voltage on C (U_C_) exceeds an upper threshold, taken as unity. (**B**) Simulated U_C_ voltage (blue line), and the corresponding burst events (grey bars). Please be aware that in panel (**B**), the scale of the positive y-axis has been magnified by a factor of 25 to enhance visibility.

**Figure 6:**
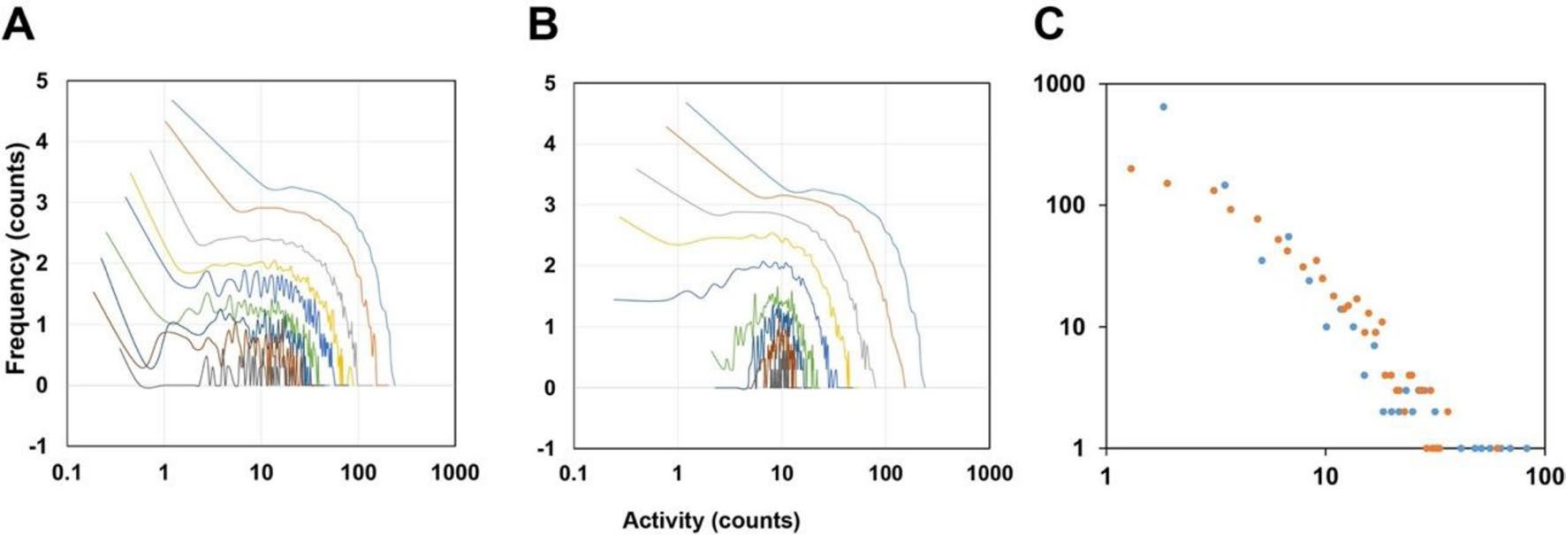
PDF-analysis of the simulated time series (**A**), and the randomized control (**B**), in a log–log representation, analogous to Fig. 2. (**C**) Distribution of active (orange symbols) and passive (blue symbols) periods in a log–log representation analogous to Fig. 3. A period was considered active if its average amplitude for a 5-min time window exceeded 20 counts/min, and passive if the time window did not exceed the 20 counts/min.

Stochastic models often give important clues for the interpretation of the dynamic behaviour of complex systems^31,34,42^. However, a simple stochastic model that could account for the main observed features of human daily activity patterns until the moment has not been elaborated. We attempted to fill this gap by applying a modified noisy integrate-and-fire model, like the one applied for the simulation of stochastic features of other typical physiological signals, such as neuron firing or heart rhythms, as described in^31,43^. In a schematic, electronic representation of the model, a random noise (δ) is superimposed to a constant drift current (I_t_) that is charging a capacitance (C), whose potential is short-cut by a discharge device (Fig. 5A), if it exceeds a threshold level. Here, the drift current represents the “inner drive” leading to an elementary action (wrist motion), while the noise accounts for natural fluctuations, mainly due to the variable internal and external conditions. Since the original models using reflective boundary conditions^31,43^ at the starting (“zero-current”) level were not able to reproduce the statistical properties of the measured actigraphy signals, we released the lower-bound conditions and allowed negative values for the driving current, as well, which may correspond to a sort of inhibition, similar to the hyperpolarization in nerve cells, in a lower level of hierarchy. Figure 5B demonstrates the model-generated time series of spikes, while the results of a PDF analysis of the simulated signals, carried out as described under section 1 for the real HPA recordings, are shown in Fig. 6A,B. The distribution of the corresponding active and passive periods is depicted in Fig. 6C.

A notable result is that a proper parameter set (drift current, noise, upper threshold level) of the independent variables of the model could qualitatively reproduce the main statistic features describing the temporal organization of activity and rest periods. Note that the main difference between the models for nerve^31^ or pacemaker heart cells^43^ and HPA is that the ratio of the charging current and the noise is considerably smaller in the latter case, responsible for the strongly stochastic appearance of actograms: While I_t_/σ ≈ 20 in the heart-rate variability model, it is about 0.025 in this case.

**Figure 7:**
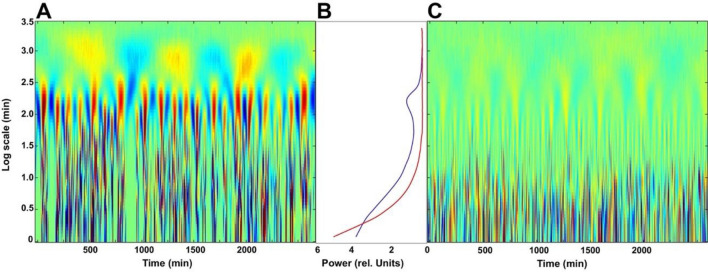
Wavelet analysis of the simulated time-series data provided by the stochastic model in Fig. 5, with considering the “mid-day pause”, as a moderate modulation of the charge current, following Fig. S4. (**A**) The colour-coded continuous-wavelet maps of the simulated activity bursts are shown, (**C**) is the same for the randomized time series. (**B**) Shows the corresponding structuredness parameter as a function of the time window (i.e., the distribution of the square-power of the wavelet coefficients over the scale dimension).

The wavelet analysis of the simulated time series of bursts also yielded similar structures at the narrow time-window ranges (Range 1 and 2 for time windows between 1 to 10 min, and 10 min to 50 min, respectively) to those of the measured actigraphy signals (Fig. 4), but the superstructure at broader time windows (Range 3 for the hours’ scale) was not reproduced by this stochastic model (not shown). When, however, the so-called “mid-day pause”, a well-known feature of typical daily actigraphy signals was considered by incorporating a moderate “afternoon dip” in the drift current (Fig. S4), the higher-order hierarchy of the typical, real-life actigraphy signals could be procreated (Fig. 7A–C), implying that the superstructure represented by the wavelet-map in Range 3 is a consequence of an “external” schedule-forming factor (not inherent in the simple stochastic model). The circadian pattern reflected in the superstructure of Range 4 is arguably another manifestation of external constraints, due to the circadian rhythm.

It is interesting to note that a fluctuation analysis of the New York Stock Exchange data revealed a similar transition from internal to external control between the time scale of minutes and the daily scale^44^, which may be a manifestation of general laws determining the dynamics of various complex systems^45^.

## Conclusions and outlook

The above findings imply an intrinsically stochastic and hierarchical structure of the organization of human daytime psychomotor activity on the time scale of minutes to hours, and beyond. The results are perfectly in line with the conclusions of the study of Barabási et al.^34^, where a statistical analysis of the timing of e-mail communications has revealed a similar ‘bursty’ nature, suggested to be a general feature of human actions, and interpreted as a fundamental consequence of decision-making. Later studies provided evidence for the bursty nature of other human actions, as well, like letter-based communications, web browsing, library visits and stock trading^42^. Our study contextualizes these conclusions within a broader framework, and extends their applicability to significantly shorter time scales. Benchmark behavioural studies have assumed that in the sequential structure of routine, everyday tasks there must be a pyramidal hierarchy of structural units of increasing duration and complexity, serving a corresponding hierarchy of purposes^46,47^. The hierarchical levels revealed by our analysis strongly support these hypotheses and are considered to provide important clues together with quantitative data for neurophysiologists developing novel brain and behavioural models^47,48^.

One might suspect, on the other hand, that the described features are not restricted to HPA, but also appear in other physiological time series. In fact, the time course of heartbeat frequency of healthy human subjects also shows a hierarchical (multifractal) structure, that is lost in case of severe heart diseases^49^, and it has been assigned to an intrinsic multiscale regulatory mechanism^16^. Analogously, the stochastic nature and hierarchical structure of HPA signals are also expected to have an adaptive function for optimizing human performance in a complex, dynamically varying environment^14^. However, an essential difference in these two time series is that the rhythmic appearance of heart-rate signals is much more emphasized than that of daytime activity signals, which appear to be much more stochastic, and it is reflected by the different ratio of the charging and the noise currents in the respective integrate-and-fire models. Also, while heart rhythms are mainly controlled by the autonomic (involuntary) nervous system, daytime activities on this time scale are expected to be dominated by conscious control^50,51^. For a deeper understanding of the effects of autonomic nervous system and voluntary actions in human motion analysis, a combined ECG-actigraphy study is in progress.

Nevertheless, already at the present state of knowledge, it is reasonable to assume that malfunctions of the cognitive mechanism should cause alterations of HPA. In fact, in case of mental disorders, like schizophrenia, bipolar psychosis, or action disorganization syndrome, characteristic anomalies in the activity patterns have been reported^41,47,52–54^. Stress has also been shown to disorganize rhythms on the ultradian scale and beyond, in various organisms including humans^55,56^. Given these premises, the utilization of the sophisticated statistical methods employed in our analysis is anticipated to markedly enhance the diagnostic methodology for psychiatric and neurological disorders^41^.

## Methods

### Data collection

For monitoring human physical activity, a MicroMini Motionlogger actigraph (Ambulatory Monitoring Inc., Ardsley NY) was used in proportional integrating mode, with 1 min/epoch time.

### Subjects

Data collected from 4 Caucasian male volunteers under daily and nightly routine conditions for an average of 3 weeks all agree on their main features. Their age was 21, 43, 49 and 51 years in ascending order, all graduated from high school or university. Occupation-wise, intellectual freelancers with no strict work time schedule were selected so as to minimize external constraints. The basic concepts are shown via the analysis of a data set recorded on a single subject (one of the above 4, from age of 51 to 52 years) for 600 days. The study was approved by the Ethics Committee of the Medical Research Council (ETT-TUKEB) operating as a board of the Ministry of Human Capacities of Hungary (approval identification No.: 15239–5/2023/EÜIG). All subjects gave prior written informed consent to participate. The study was carried out according to the Declarations of Helsinki and the standards set forth by the journal for ethical human research^57^.

### PDF analysis

For probability density function analysis, successive daytime (awake) activity recordings were concatenated into one time series, whose time scale was divided into equal intervals (“boxes”), and activity values were averaged within each box, respectively. PDF functions were calculated from these averaged values for some characteristic box lengths between 1 and 720 min (Fig. S1). In order to calculate the lengths of active and passive periods, the average activity of the original recordings was determined over a 5-min, shifting time window. A period was considered active if its average activity was higher than 20 counts/min, and passive otherwise. Both PDFs were fitted by lognormal distribution of the positive random variable, x, defined by the conventional μ and σ parameters (mean and square root of variance, respectively):

$$y = f\left( {x|\mu ,\sigma } \right) = \frac{1}{{x\sigma \sqrt {2\pi } }}e^{{\frac{{ - (\ln x - \mu )^{2} }}{{2\sigma^{2} }}}}$$

All the numerical calculations were carried out by the MATLAB program (MathWorks Inc., Natick, MA).

### Wavelet transform

To visualize the typical multiscale pattern structure of the actograms we calculated the continuous wavelet transform (CWT) of the actograms. In order to investigate the awake and sleep data separately, daily awake and sleep periods were identified by applying a common ad-hoc threshold (10 counts/min for a 20-min sliding window), and then 10 successive awake or sleep activity recordings were concatenated into one daytime and one nocturnal time series, respectively.

The CWT of a time-dependent signal f(t) is defined by Mallat^36^

$$c(u,s) = \int\limits_{ - \infty }^{\infty } {f(t)\frac{1}{\sqrt s }} \Psi^{*} \left( {\frac{t - u}{s}} \right)dt,$$

where $\Psi (t)$ is the wavelet function, the transform is based on and * denotes complex conjugate. The resulting wavelet coefficients $c(u,s)$ define a 2-dimensional function depending on the translation parameter *u*, mapped to the original time variable *t*, and a scale parameter *s*. The particular transform applied to the actogram was based on the Morlet wavelet^57^

$$g(t) = \frac{1}{{\sqrt {\pi \gamma_{b} } }}e^{{ - \frac{{t^{2} }}{{\gamma_{b} }}}} e^{{ - i\omega_{c} t}} ,$$

where $\omega_{c} /2\pi$ corresponds to the center frequency and $\gamma_{b}$ to the bandwidth of the transform. With a time unit of 1 min, we found that the parameter values of $\omega_{c} = 10$ and $\gamma_{b} = 2$ resulted in a good tradeoff between the time and scale resolution of the characteristic patterns occurring in the actograms, hence we applied these values in all calculations. In the presented figures and Fig. S3 the images created from the real part of the complex coefficients are shown (the imaginary part yields very similar pictures). This representation visualizes better the pattern structure of the data than the corresponding absolute values and phases. To avoid the dominance of the day-night periodicity in the activity signals, the CWT was calculated on a dataset built up by concatenation of the 802-min wakeful periods of 3 subsequent days. The scale variable *s* spanned logarithmically the range of 10^0.8^ to 10^3.15^. Expressed in the term of harmonics, with the value of $\omega_{c} = 10$, the corresponding limits in time of period units are ~ 4 min and ~ 888 min, respectively. The higher limit safely exceeds the length of a single wakeful period.

In the definition of CWT the factor of $1/\sqrt s$ was introduced for normalization purposes. However, this is resulted in lower weights to the pattern components corresponding to lower scales, since an equal weight would require a factor of $1/s$. For this reason, to achieve a more balanced visual presentation, in Fig. 4 and Fig. S3 the calculated wavelet coefficients were additionally divided by $\sqrt s$.

We introduced a ‘structuredness’ parameter, P(s), for the distribution of the power of the CWT coefficients on the scale variable, calculated according to

$$P(s) = \int\limits_{u} {abs\left( {c\left( {s,u} \right)} \right)}^{2} du.$$

To obtain smooth curves, P(s) was determined from the concatenated wakeful periods of 10 subsequent days.

The numerical calculation of the wavelet transforms was carried out in the Wavelet Toolbox of the MATLAB program (MathWorks Inc., Natick, MA).

### Supplementary Information

Supplementary Figures.

## Data Availability

The datasets used and/or analyzed during the current study available from the corresponding author on reasonable request.

## References

[1] Maczak B, Vadai G, Der A, Szendi I, Gingl Z (2021). Detailed analysis and comparison of different activity metrics. PLoS One.

[2] Fekedulegn D (2020). Actigraphy-based assessment of sleep parameters. Ann. Work Expo Health.

[3] Leuenberger, K. D. *Long-Term Activity and Movement Monitoring in Neurological Patients* (ETH Zürich, 2015).

[4] Faedda GL (2016). Actigraph measures discriminate pediatric bipolar disorder from attention-deficit/hyperactivity disorder and typically developing controls. J. Child Psychol. Psychiatry.

[5] Ancoli-Israel S (2003). The role of actigraphy in the study of sleep and circadian rhythms. Sleep.

[6] Turing AM (1990). The chemical basis of morphogenesis. Bull. Math. Biol..

[7] Gierer A, Meinhardt H (1972). A theory of biological pattern formation. Kybernetik.

[8] Zhadin MN (2001). Review of Russian literature on biological action of DC and low-frequency AC magnetic fields. Bioelectromagnetics.

[9] Blessing W, Ootsuka Y (2016). Timing of activities of daily life is jaggy: How episodic ultradian changes in body and brain temperature are integrated into this process. Temperature (Austin).

[10] Gerkema MP, Kumar V (2002). Ultradian rhythms. Biological Rhythms.

[11] Aschoff, J. C. & Gerkema, M. P. On Diversity and Uniformity of Ultradian Rhythms (1985).

[12] Daan, S. & Aschoff, J.C. Short-Term Rhythms in Activity. (1981).

[13] Brodsky VY (2014). Circahoralian (ultradian) metabolic rhythms. Biochemistry (Mosc).

[14] Lloyd D, Stupfel M (1991). The occurrence and functions of ultradian rhythms. Biol. Rev..

[15] Glass L (2001). Synchronization and rhythmic processes in physiology. Nature.

[16] Hu K (2004). Non-random fluctuations and multi-scale dynamics regulation of human activity. Physica A.

[17] Amaral LAN (2004). Power law temporal auto-correlations in day-long records of human physical activity and their alteration with disease. Europhys. Lett..

[18] Rossi, E. L. & Nimmons, D. (1991) The 20-Minute Break: Using the New Science of Ultradian Rhythms. Los Angeles: Jeremy P. Tarcher, Inc. Reviewed by: George Gafner, LCSW, Southern Arizona Veterans Affairs Health Care System, Tucson, AZ. *Am. J. Clin. Hypnosis* **48**, 217–218 (2005).

[19] Young, E. & Korszun, A. Stress, the HPA axis and depressive illness (2009).

[20] Kleitman N (1982). Basic rest-activity cycle–22 years later. Sleep.

[21] Gerkema MP, Groos GA, Daan S (1990). Differential elimination of circadian and ultradian rhythmicity by hypothalamic lesions in the common vole, *Microtus*
*arvalis*. J. Biol. Rhythms.

[22] Prendergast BJ, Zucker I (2016). Ultradian rhythms in mammalian physiology and behavior. Curr. Opin. Neurobiol..

[23] Goh GH, Maloney SK, Mark PJ, Blache D (2019). Episodic ultradian events-ultradian rhythms. Biology (Basel).

[24] Ptitsyna NG, Villoresi G, Dorman LI, Iucci N, Marta IT (1998). Natural and man-made low-frequency magnetic fields as a potential health hazard. Physics-Uspekhi.

[25] Diatroptov ME, Arseniev GN, Ligun NV, Diatroptova MA, Dorokhov VB (2023). Effect of heliogeophysical and atmospheric factors on the degree of synchronization of ultradian rhythms of body temperature in mice. Bull. Exp. Biol. Med..

[26] Maczak B, Gingl Z, Vadai G (2024). General spectral characteristics of human activity and its inherent scale-free fluctuations. Sci. Rep..

[27] Zacks JM (2001). Human brain activity time-locked to perceptual event boundaries. Nat. Neurosci..

[28] Limpert E, Stahel WA, Abbt M (2001). Log-normal Distributions across the Sciences: Keys and Clues: On the charms of statistics, and how mechanical models resembling gambling machines offer a link to a handy way to characterize log-normal distributions, which can provide deeper insight into variability and probability—normal or log-normal: That is the question. BioScience.

[29] Dehaene S, Kerszberg M, Changeux J-P (1998). A neuronal model of a global workspace in effortful cognitive tasks. Proc. Natl. Acad. Sci..

[30] Sackur J, Dehaene S (2009). The cognitive architecture for chaining of two mental operations. Cognition.

[31] Kish EA, Granqvist CG, Der A, Kish LB (2015). Lognormal distribution of firing time and rate from a single neuron?. Cogn. Neurodyn..

[32] Buzsáki G, Mizuseki K (2014). The log-dynamic brain: How skewed distributions affect network operations. Nat. Rev. Neurosci..

[33] Scheler G (2017). Logarithmic distributions prove that intrinsic learning is Hebbian. F1000Res.

[34] Barabasi AL (2005). The origin of bursts and heavy tails in human dynamics. Nature.

[35] Nakamura T (2007). Universal scaling law in human behavioral organization. Phys. Rev. Lett..

[36] Mallat S (1999). A Wavelet Tour of Signal Processing.

[37] Dehaene S, Changeux JP, Naccache L, Sackur J, Sergent C (2006). Conscious, preconscious, and subliminal processing: A testable taxonomy. Trends Cogn. Sci..

[38] Wehr TA (1998). Treatment of rapidly cycling bipolar patient by using extended bed rest and darkness to stabilize the timing and duration of sleep. Biol. Psychiatry.

[39] Xavier WDS (2024). The sleep patterns of children and adolescents with chronic conditions and their families: An integrative literature review. Children.

[40] Ibanez V, Silva J, Cauli O (2018). A survey on sleep assessment methods. PeerJ.

[41] Nagy Á (2023). The actigraphy-based identification of premorbid latent liability of schizophrenia and bipolar disorder. Sensors.

[42] Vázquez A (2006). Modeling bursts and heavy tails in human dynamics. Phys. Rev. E.

[43] Büzás A, Horváth T, Dér A (2022). A novel approach in heart-rate-variability analysis based on modified Poincaré plots. IEEE Access.

[44] Eisler Z, Kertész J, Yook SH, Barabási AL (2005). Multiscaling and non-universality in fluctuations of driven complex systems. Europhys. Lett..

[45] Eisler Z, Bartos I, Kertész J (2008). Fluctuation scaling in complex systems: Taylor's law and beyond. Adv. Phys..

[46] Fuster JM, Holstege G (1991). Chapter 10 The prefrontal cortex and its relation to behavior. Progress in Brain Research.

[47] Botvinick M, Plaut DC (2004). Doing without schema hierarchies: A recurrent connectionist approach to normal and impaired routine sequential action. Psychol. Rev..

[48] Zylberberg A, Dehaene S, Mindlin G, Sigman M (2009). Neurophysiological bases of exponential sensory decay and top-down memory retrieval: A model. Front. Comput. Neurosci..

[49] Goldberger AL (2002). Fractal dynamics in physiology: Alterations with disease and aging. Proc. Natl. Acad. Sci. USA.

[50] Pujol J (2021). Largest scale dissociation of brain activity at propofol-induced loss of consciousness. Sleep.

[51] Buzsaki G (2015). Hippocampal sharp wave-ripple: A cognitive biomarker for episodic memory and planning. Hippocampus.

[52] Haug HJ, Wirz-Justice A, Rossler W (2000). Actigraphy to measure day structure as a therapeutic variable in the treatment of schizophrenic patients. Acta Psychiatr. Scand. Suppl..

[53] Dancshazy Z (2004). Phase-synchronization of daily motor activities can reveal differential circadian patterns. Chronobiol. Int..

[54] Krane-Gartiser K, Henriksen TE, Morken G, Vaaler A, Fasmer OB (2014). Actigraphic assessment of motor activity in acutely admitted inpatients with bipolar disorder. PLoS One.

[55] Harper DG, Tornatzky W, Miczek KA (1996). Stress induced disorganization of circadian and ultradian rhythms: Comparisons of effects of surgery and social stress. Physiol. Behav..

[56] Erdei L (1998). Environmental stress and the biological clock in plants: Changes of rhythmic behavior of carbohydrates, antioxidant enzymes and stomatal resistance by salinity. J. Plant Physiol..

[57] Teolis A (1998). Computational Signal Processing with Wavelets.

