# Supplementary material for: Hierarchical organization of human physical activity
Source: Sci Rep. 2024 Mar 12;14:5981. doi: 10.1038/s41598-024-56185-0 (PMC10933410; doi:10.1038/s41598-024-56185-0)
Supplement: Supplementary file 1 — Supplementary Figures. [file 41598_2024_56185_MOESM1_ESM.docx]

Supplementary Online Content for the paper:

Hierarchical Organization of Human Physical Activity

András Búzás^1^, András Makai^1^, Géza I. Groma^1^, Zsolt Dancsházy^1†^, István Szendi^2^, Laszlo B. Kish^3^, Ana Raquel Santa-Maria^1,4*^, András Dér^1*^

^1^Institute of Biophysics, HUN-REN Biological Research Centre, H-6701 Szeged, Temesvári krt. 62, P.O.B. 521

^2^Department of Psychiatry, Kiskunhalas Semmelweis Hospital, 1 Dr. Monszpart László Street, 6400 Kiskunhalas, Hungary

^3^Department of Electrical and Computer Engineering, Texas A&M University, TAMUS 3128, College Station, TX 77843-3128, USA

^4^ Wyss Institute for Biologically Inspired Engineering, Harvard University, Boston, Massachusetts, USA

^†^deceased

^*^To whom correspondence should be addressed. Email: anaraquel.santamaria@wyss.harvard.edu, der.andras@brc.hu


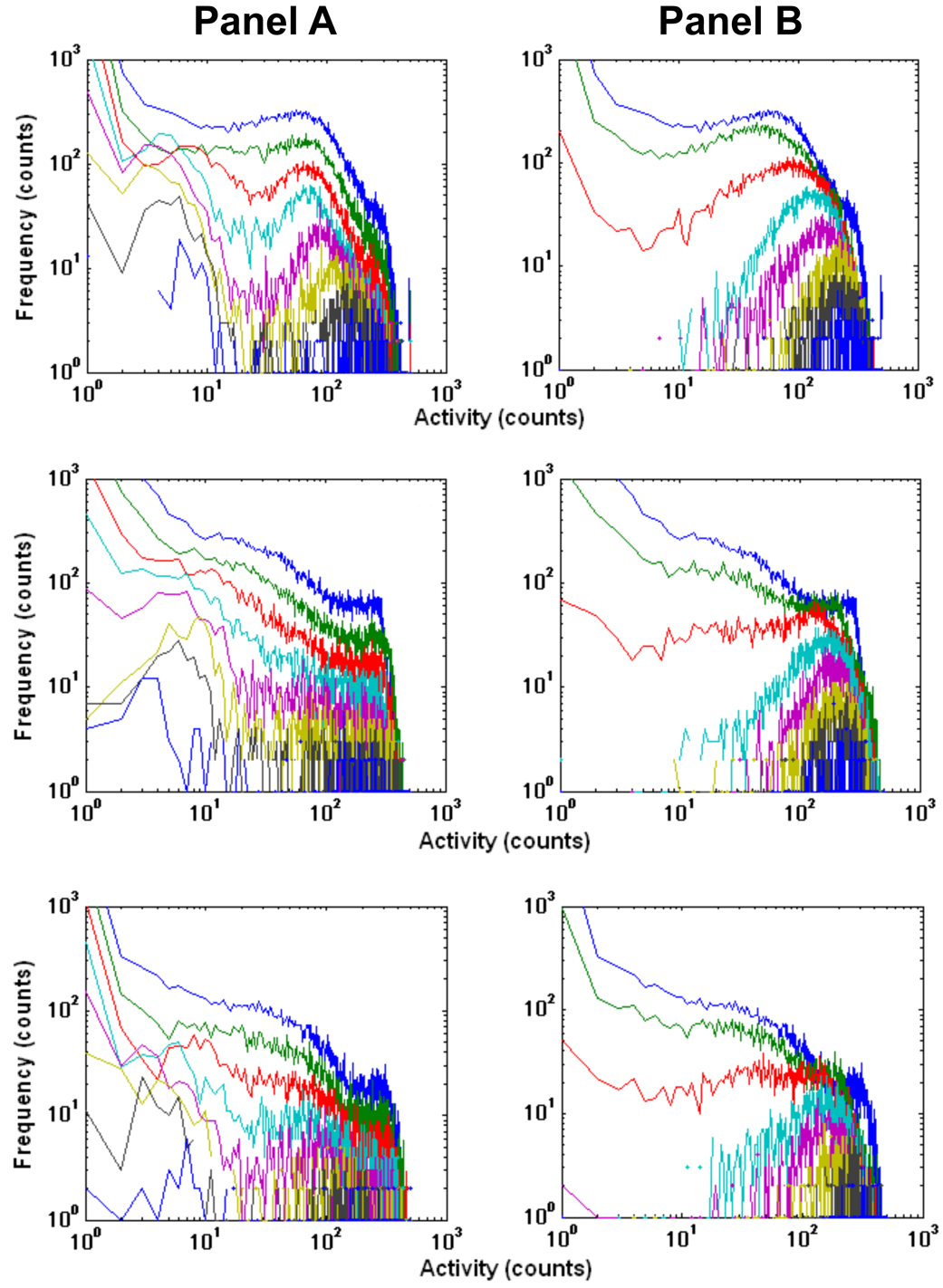


**Figure S1.**  Panel A, The PDF of the daytime activity counts from data of the 3 additional volunteers, as a function of time resolution (“box length”). Panel B, The same PDF analysis of a virtual time series got by randomly mixing the original recording data in time (scrambling). Data are shown for some characteristic, quasi-exponentially distributed box lengths (1, 2, 5, 11, 22, 45, 90, 180, 360 and 720 minutes) distinguished by different colors (blue, green, red, cyan, magenta, yellow, black, blue, green, red), respectively. Increasing the box size above 240 min, results in the disappearance of one of the two peaks. At the same time the remaining peak is centered in between the former two (closer to the higher value) (panel A, lowest curve), corresponding to a weighted averaging of high and low activities at this box length scale. From here on, the center of this peak does not change significantly in the time range studied, and practically corresponds to the average daytime activity.

**
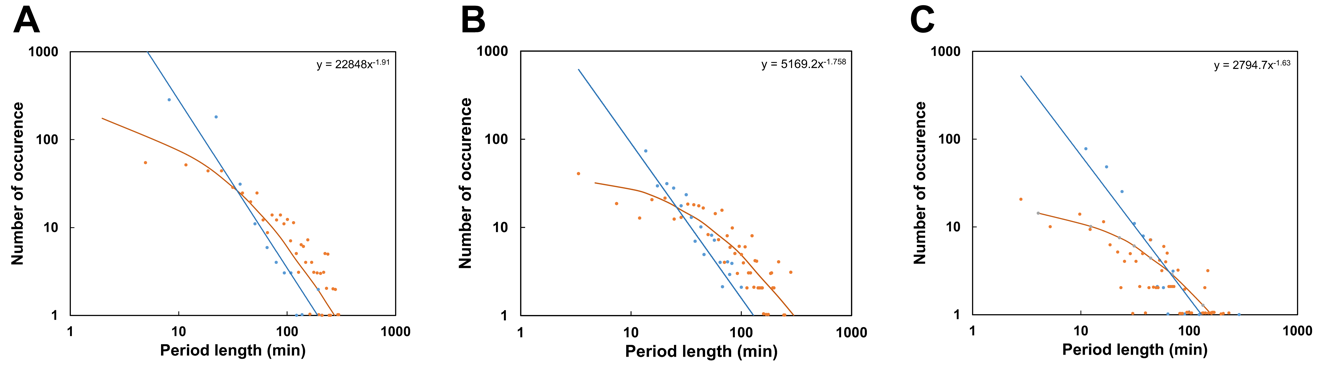
**

**Figure S2.** Probability density functions of the lengths of the daytime active and passive periods (red and blue symbols, respectively) in the original recordings of the 3 additional volunteers. PDFs of active periods were fitted by lognormal distribution defined by the conventional μ and σ parameters. The average length of active periods is considerably higher. However, periods longer than 50 mins are rare, also for this case. The probability of periods longer than 4 hours is less than 10^-3^. Note, that although the actual parameters of the distributions of active and passive periods were found to differ for different subjects (3.0 ≤ μ_act_ ≤ 4.0 and 1.3 ≤ σ_act_ ≤ 1.6 for active periods, while slopes -2 < -1.5 for passive periods), the overall features of the PDF-s remained the same.


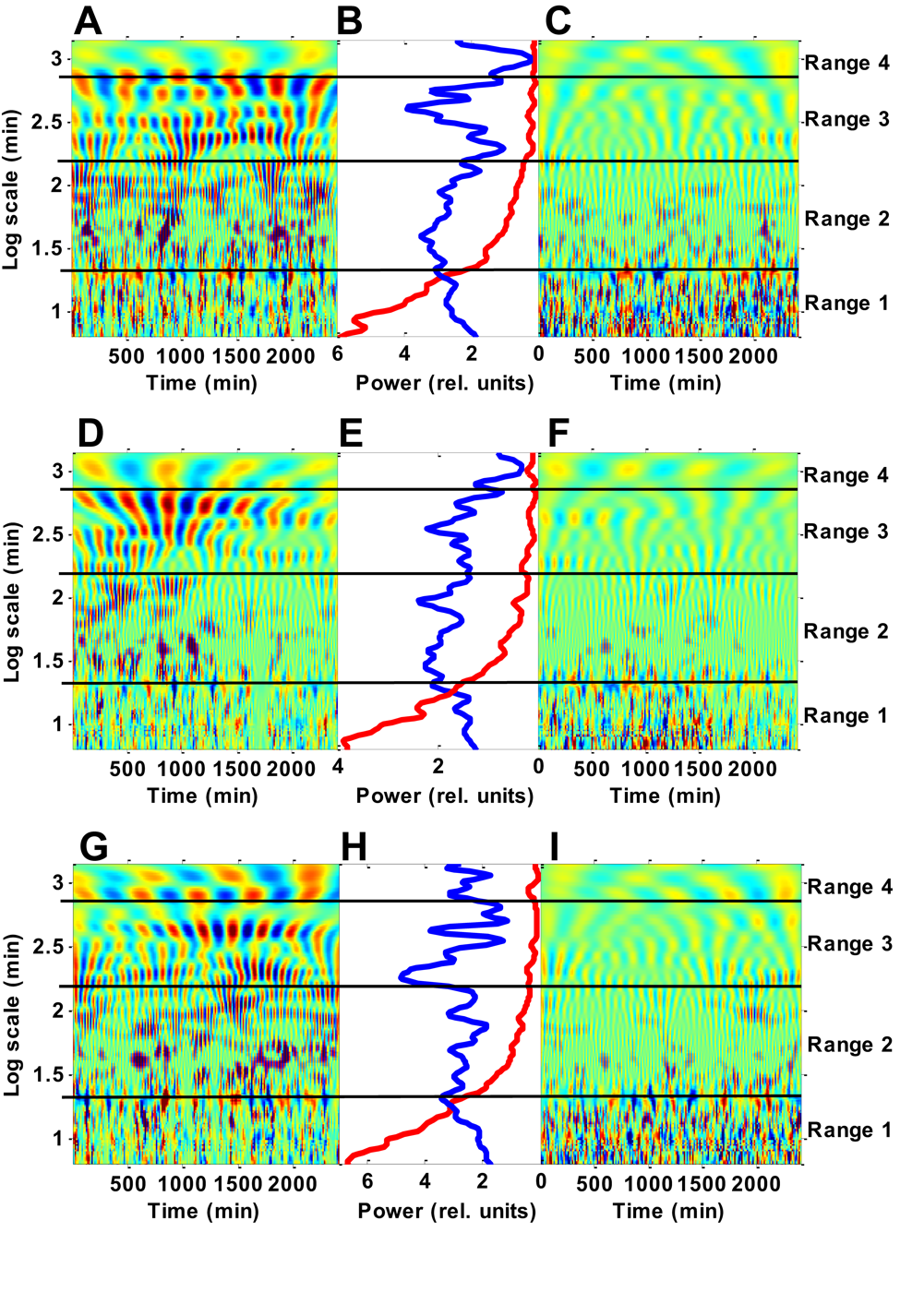


**Figure S3.** Wavelet representation of the daytime actograms taken from the 3 additional volunteers (A-C, D-F and G-I, respectively). Data processing and the method of presentation are identical to that applied to Fig. 4 of the main text. For better comparison, the positions of the linear bars separating the typical ranges are also inherited from that figure.


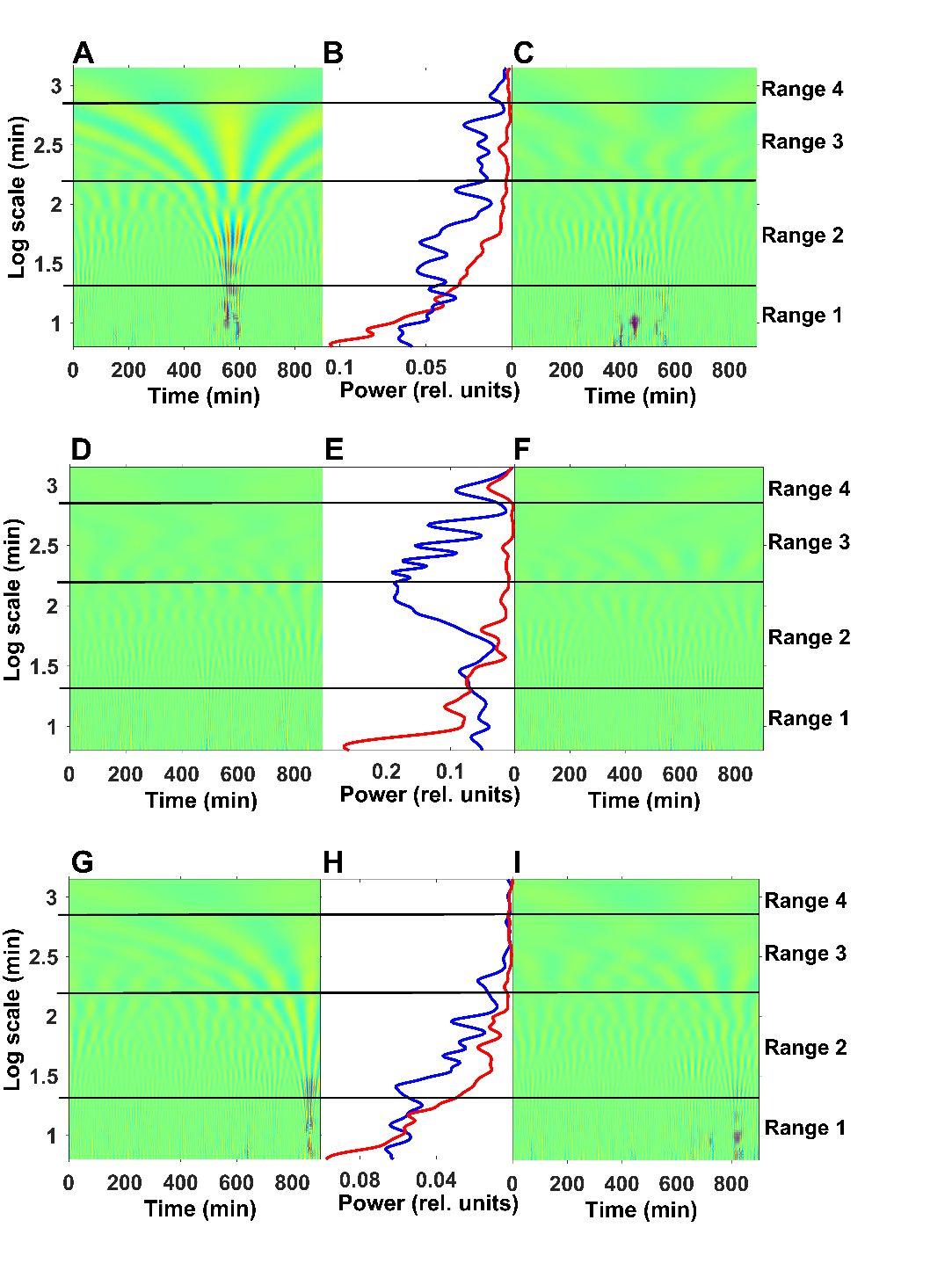


**Figure S4.** Wavelet representation of the nocturnal (sleep) actograms taken from the 3 additional volunteers (A-C, D-F and G-I, respectively). Data processing and the method of presentation are identical to that applied to Fig. 4 of the main text. For better comparison, the positions of the linear bars separating the typical ranges are also inherited from that figure.

**Figure S5.** The average of 233 consecutive daily actograms, synchronized to the calendar hours. Note the afternoon dip between ca. 12h and 15h.
